# Glassy carbon microelectrodes minimize induced voltages, mechanical vibrations, and artifacts in magnetic resonance imaging

**DOI:** 10.1038/s41378-019-0106-x

**Published:** 2019-11-18

**Authors:** Surabhi Nimbalkar, Erwin Fuhrer, Pedro Silva, Tri Nguyen, Martin Sereno, Sam Kassegne, Jan Korvink

**Affiliations:** 1 MEMS Research Lab, Department of Mechanical Engineering,College of Engineering, San Diego State University, 5500 Campanile Drive, San Diego, CA 92182 USA; 2 NSF-ERC Center for Neurotechnology (CNT), Seattle, WA USA; 3 Institute of Microstructure Technology – Karlsruhe Institute of Technology, Hermann-von-Helmholtz-Platz, 76344 Eggenstein-Leopoldshafen, Germany; 4 Magnetic Resonance Imaging Lab, San Diego State University, San Diego, CA 92182 USA

**Keywords:** Electronic properties and materials, Electrical and electronic engineering

## Abstract

The recent introduction of glassy carbon (GC) microstructures supported on flexible polymeric substrates has motivated the adoption of GC in a variety of implantable and wearable devices. Neural probes such as electrocorticography and penetrating shanks with GC microelectrode arrays used for neural signal recording and electrical stimulation are among the first beneficiaries of this technology. With the expected proliferation of these neural probes and potential clinical adoption, the magnetic resonance imaging (MRI) compatibility of GC microstructures needs to be established to help validate this potential in clinical settings. Here, we present GC microelectrodes and microstructures—fabricated through the carbon micro-electro-mechanical systems process and supported on flexible polymeric substrates—and carry out experimental measurements of induced vibrations, eddy currents, and artifacts. Through induced vibration, induced voltage, and MRI experiments and finite element modeling, we compared the performances of these GC microelectrodes against those of conventional thin-film platinum (Pt) microelectrodes and established that GC microelectrodes demonstrate superior magnetic resonance compatibility over standard metal thin-film microelectrodes. Specifically, we demonstrated that GC microelectrodes experienced no considerable vibration deflection amplitudes and minimal induced currents, while Pt microelectrodes had significantly larger currents. We also showed that because of their low magnetic susceptibility and lower conductivity, the GC microelectrodes caused almost no susceptibility shift artifacts and no eddy-current-induced artifacts compared to Pt microelectrodes. Taken together, the experimental, theoretical, and finite element modeling establish that GC microelectrodes exhibit significant MRI compatibility, hence demonstrating clear clinical advantages over current conventional thin-film materials, further opening avenues for wider adoption of GC microelectrodes in chronic clinical applications.

## Carbon microstructures: MRI-friendly neural implants

Evidence suggests that neural implants based on glassy carbon are safe during MRI scans and do not distort scan images. Glassy carbon (GC) is becoming a preferential material in biosensors such as implants for brain wave recording or deep brain stimulation. To be useful, new neural implants should be maintained compatible with MRI scanning, as MRI has previously been known to induce vibration and heating of implants, induce an electrical current in the implant, and distort MR images. Sam Kassegne’s team at San Diego State University & Center for Neurotechnology (CNT) collaborated with Jan Korvink’s team at Karlsruhe Institute of Technology in Germany and pitted GC microelectrodes against conventional platinum microelectrodes. GC microelectrodes exhibited superior compatibility with MRI scanning compared to platinum, producing no significant vibration or induced current, yet were still visible in the images. The team expects further research will find that GC microelectrodes do not significantly heat during MRI scanning.

## Introduction

Increasingly, carbon is becoming a compelling material of choice for the micro- and nanofabrication of a variety of micro devices with applications varying from biochemical sensors to microcapacitors and batteries^1–3^. The recent introduction of neural probes consisting of glassy carbon (GC) microelectrodes microfabricated through carbon micro-electro-mechanical systems (C-MEMS) technology and transferred to flexible polymer substrates has opened up significant opportunities in wearable and implantable carbon devices. This trend will continue as more evidence supporting the superior performance of GC microstructures in applications requiring extended electrical, electrochemical, and mechanical stability under chronic in vivo conditions emerge^4^.

In the meantime, magnetic resonance imaging (MRI) is increasingly being used under pre- and post-surgery conditions for the brain imaging of patients as well as animal models already implanted with electrocorticography (ECoG) or deep brain stimulation (DBS)-type neural probes. These MRI modalities are typically needed to investigate outcomes of DBS or chronic sulcus electrode placement and to evaluate pathological abnormalities related to electrode implantation^5^. They are also used for guiding surgery in chronically implanted microelectrode arrays in inaccessible areas of the cortical sulcus where a magnetization-prepared rapid acquisition with gradient-echo (MPRAGE) sequence is acquired for stereotaxically positioning the array^6^. In addition, the 3D atlas MRI co-registration method has been used to localize stimulating electrodes of DBS systems in Parkinson’s disease patients^7^.

However, there is a significant number of documented cases where neural implants give rise to visible artifacts in MRI images^8^. Specifically, these MR images have shown distortion artifacts due to mismatches in the magnetic susceptibility of electrode materials and brain tissue, leading to inaccurate observations of electrodes in MR images due to hypo or hyper signals at these interfaces^7^. Furthermore, the interaction between conductive materials and radio frequency (RF) fields results in the heating of electrode surfaces, in which the transmitted RF field is absorbed in the electrode material, causing heating^9,10^. An increase in temperature up to 7 °C due to RF electrical field induction of current in the leads of electrodes has been reported^11^. In addition, since neural implant materials are usually tested under high magnetic fields (up to 3 T), excitation by RF and rapidly switching gradients cause not only imaging artifacts but also eddy currents. The use of MRI, therefore, understandably raises concerns about risks such as heating of the cortex due to induced currents^11,12^ and mechanical movements of electrodes due to gradient-induced vibrations.

In general, while designing cortical electrode arrays compatible with MRI, the hazards to be considered are (1) forces generated due to the interaction between gradient fields and the static *B*_0_ field with the implanted electrode, (2) induced voltages due to a changing gradient field and RF field and movement of the electrode inside the scanner, (3) heating of the electrode due to RF pulses and electric fields generated by a time-varying magnetic field, and (4) imaging artifacts originating from (a) susceptibility mismatch between the electrode material and surrounding brain tissue and/or (b) *B*_1_-field distortion due to induction^8^. To avoid or minimize the imaging artifacts, electrode materials with magnetic susceptibility matching that of brain tissue have been introduced, such as printed carbon ink electrodes on organic polymer substrates^13^, titanium-based microelectrodes^14^, silicon microelectrodes for neural recording and stimulations^15^, and carbon nanotube yarn electrodes^16^. However, while these materials have good MRI compatibility, they nonetheless suffer from a lack of long-term electrical and electrochemical stability. In this study, therefore, we build upon the excellent magnetic susceptibility performance of carbon materials and the excellent stability of GC^4^ and investigate the response of GC microelectrodes to MRI from the perspective of induced voltages, mechanical forces, and vibrations, as well as artifacts. We additionally create a finite element model (FEM) to validate the experimental measurements of induced voltages, currents, and mechanical forces. The aim is, therefore, to extend the application of GC to implantable devices, where other modalities such as live and simultaneous MRI are required for clinical purposes on subjects with implanted neural devices.

## Materials and methods

### Microfabrication of GC microelectrodes

**Fig. 1:**
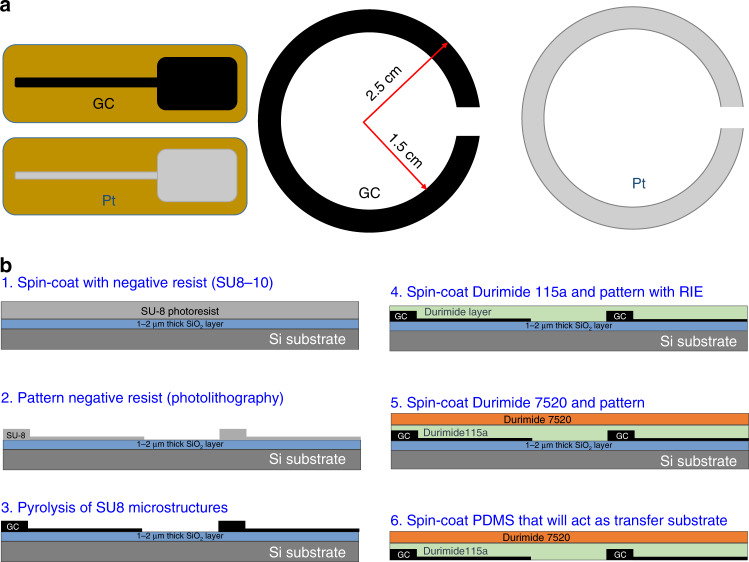
Test eletrodes for probe characterisation. **a** Probes with GC and Pt ground microelectrodes with a geometry of 2 cm × 1 cm supported on a polyimide substrate, and circular GC and Pt microelectrodes (OD = 5 cm, ID = 3 cm) supported on a silicon substrate. **b** UV photolithography steps for microfabrication of GC microelectrodes on a flexible substrate

To investigate and compare the MRI compatibility of GC microelectrodes and thin-film metal microelectrodes, we fabricated probes made of both GC and Pt microelectrodes in two sets as shown in Fig. 1. The first set consisted of a ground electrode of an ECoG probe (one with GC and another with Pt with a rectangular geometry of 2 cm × 1 cm) supported on a polymer substrate, while the second set consisted of ring electrodes of 5 cm outer diameter and 3 cm internal diameter supported on a silicon wafer. Ground microelectrodes were selected for the MRI study because they represented microelectrodes with the largest surface area in an array of recording and stimulation electrodes in neural probes. The core C-MEMS microfabrication technology that is used for these GC devices supported on polymeric substrates is described in detail elsewhere^17^. Briefly, a SU8-10-negative photoresist (Microchem, Westborough, MA, USA) with a final thickness of 6 μm was spin coated, and a ground microelectrode (2 cm × 1 cm) was patterned. Pyrolysis of the negative resist layer was conducted following protocols described elsewhere^18,19^. In brief, the pyrolysis of the patterned microelectrode array was carried out in a quartz furnace under a vacuum or an inert atmosphere of nitrogen gas with a flow of 50 ml/min at 1000 °C for 90 min. The ensuing GC layer was then covered with a layer of non-photosensitive Durimide 115a and patterned (protocol described elsewhere)^4^. Subsequently, a thicker layer of Durimide 7520 (Fujifilm USA, Inc., Mesa, AZ, USA) was photolithographically patterned to provide a stiffer substrate for the ground microelectrode. This was followed by etching of the silicon dioxide in a buffered hydrofluoric acid bath. For the probes with Pt ground microelectrodes, conventional metal lift-off on a polymer layer was carried out. The details are given in the Supplementary Section (Fig. S1).

### MRI measurements2

**Fig. 2:**
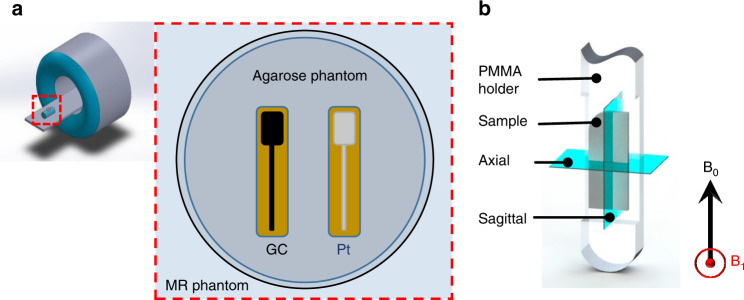
Arrangement of test probes in the MRI scanner. **a** 3 T Human MRI scanner with glassy carbon and platinum microelectrodes placed on an MRI phantom and GC and Pt cortical microelectrodes immersed in agarose; (**b**) quarter of a microelectrode specimen mounted in an 11.7 T scanner

The MR images of GC and Pt microelectrodes of identical dimensions (2 cm × 1 cm) shown in Fig. 1a were acquired by a Siemens 3 T Prisma scanner that is commonly used for clinical imaging (Siemens GmbH, Erlangen, Germany). To simulate the brain tissue environment, a phantom was prepared by dissolving phosphate-buffered saline (PBS, 0.01 M) in agarose in a glass Petri dish, where the GC and Pt microelectrodes were subsequently immersed. The agarose gel solution was allowed to solidify to avoid the trapping of bubbles. As shown in Fig. 2, the agarose phantom with the microelectrodes was then placed on top of the MRI phantom (solution N per 1000 g H_2_O dist.: 3.75 g NiSO_4_ × 6H_2_O, 5 g NaCl, 5300 ml, Siemens Healthcare GmbH, Germany) to position the microelectrode near the isocenter of the coil. MR images of the PBS-agarose phantom with GC and Pt microelectrodes were collected to test for MRI artifacts (Fig. 2a). The following MR sequences were run with the 3 T scanner: (i) T1-weighted images were acquired using an inversion-prepared 3D gradient-echo sequence (MPRAGE) with 0.8 × 0.8 × 0.8 mm^3^ resolution, flip angle = 9°, echo time (TE) = 3.15 ms, repetition time (TR) = 7.7 ms, inversion time (TI) = 900 ms, field of view (FOV) = 200 × 200 × 179 mm^3^, and inversion repeat time = 2300 ms; (ii) T2-weighted images were collected using a variable flip angle 3D turbo spin echo (TSE) sequence with 0.9 × 0.9 × 0.9 mm^3^ resolution, TE = 408 ms, TR = 3200 ms, FOV = 230 × 230 × 173 mm^3^, and averages = 1.4; (iii) a 2D dual echo *B*_0_-field map was obtained with 3 × 3 × 2 mm^3^ resolution, TE1 = 4.92 ms, TE2 = 7.38 ms, TR = 600 ms, FOV = 192 × 192 mm^2^, and flip angle = 60°; and (iv) gradient-echo multiband EPI sequence (functional MRI) images were acquired using a multiband echo planar imaging sequence with 2 × 2 × 1 mm^3^ resolution, TR = 1.02 s, TE = 31 ms, and flip angle = 60°.

Furthermore, for a higher spatial resolution, we acquired 3D gradient-echo images (GRE) and *B*_0_-field maps using an 11.7 T scanner (500 MHz Bruker Avance III, Ettlingen, Germany). Due to limited sample space, we trimmed the GC and Pt microelectrodes to quarter pieces and mounted them onto a sample holder that was placed inside the wide-bore magnet, as shown in Fig. 2b. We used a standard 3D Cartesian GRE sequence with TR = 30 ms, TE = 2.02 ms, FA = 15°, and an isotropic resolution of 100 µm. To investigate the effect of B_1_ distortion, the sample was oriented such that the *B*_1_ vector was orthogonal to the surface of the electrodes^20^. The *B*_0_-field map was acquired using a predefined integrated protocol in the machine software (ParaVision, FieldMap), which uses two GRE sequences with varying echo times. In this case, the samples were oriented such that the *B*_1_ vector was parallel to the surface of the microelectrodes to suppress *B*_1_-field distortions. The *B*_0_-field map parameters were identical to the GRE sequence except TE1 = 1.675 ms and TE2 = 26.811 ms.

### Finite element modeling of *B*_0_-field map

FEM simulations of the effects observed were modeled in COMSOL Multiphysics (COMSOL AB, Sweden) using the 2 cm × 1 cm rectangular microelectrodes as a domain (Fig. S2). Static magnetic fields (*B*_0_ effects) were obtained by solving for the magnetic scalar potential using Gauss’ law, the dynamic electromagnetic behavior (gradient effects) by solving for the magnetic vector potential and the current fields using Ampere’s and Faraday’s laws, and the radio frequency behavior (*B*_1_ effects) by solving for the electric field of the Maxwell equations. The simulated materials were Pt (*ε*_r_ ~=0.735, *χ* = 279 p.p.m., *σ* = 9.43 × 10^6^ S/m), GC (*ε*_r_ ~=12.5, *χ* = −1.2 p.p.m., *σ* = 6803 S/m), and poly(methyl methacrylate) (PMMA) (*ε*_r_ ~=2.6, *χ* = −1.2 p.p.m., *σ* ∼=0 S/m) in a water phantom (*ε*_r_ ~= 80, *χ* = −9.05 p.p.m., *σ* ∼=0 S/m)^21–24^.

### Gradient-induced vibration measurements

**Fig. 3:**
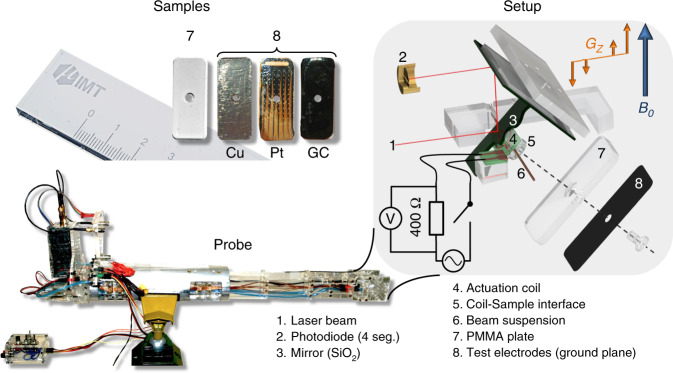
Details of the experimental setup for the measurement of gradient-induced vibrations in the field of a permanent magnet 1.5 T MRI system (ICON, Bruker, Germany). Top left: Image (with cm scale bar) of the test samples. Top right: Illustration of the measurement head. The microelectrode (8) is fixed onto a PMMA plate (7) and mounted to a coil–sample interface. The coil–sample interface is suspended on a torsion beam for which the static angular deflection is proportional to the second moment of the cross-sectional area of the beam. The vibration is recorded by measuring the deflection of a laser beam using a segmented photodiode (2). Bottom: Photograph of the complete measurement probe (Cu: copper foil, 40 µm thick; Pt: platinum microelectrode, 300 nm thick; and GC: glassy carbon microelectrode, 2 µm thick)

All vibrational experiments were conducted within a 1.5 T permanent magnet system (Bruker, ICON). A custom-built probe^25,26^ was used to investigate the torque produced by the ground microelectrode when exposed to gradient switching. The main experimental setup is illustrated in Fig. 3. Vibration occurs if conductive structures are placed inside a static magnetic field where an additional time-variable magnetic field featuring a linear gradient is superimposed. In the setup presented here, the produced torque leads to a mechanical deflection that could be precisely measured by reflection of a laser beam using a segmented photodiode.

To acquire the torque produced by different implants, we fixed the microelectrodes onto a PMMA substrate and mounted it to the coil–sample interface. Then, we followed a three-step measurement protocol to determine the torque produced by the implants. The protocol is described in Supplementary Section C.

The mechanical setup illustrated in Fig. 3 is modeled by a harmonic oscillator using Eq. 1.

1 $${J}\ddot \theta + {\Gamma }\dot \theta + \mu \theta = \tau \left( t \right),$$

where *J* is the moment of inertia, *Γ* is the rotational friction, *µ* is the torsion constant, *τ* is the external torque, and *θ* is the measured deflection angle. We designed the setup to produce the externally applied torque in two ways. The first involved a precisely controlled current *I(t)* with respect to the amplitude and frequency that was passed through an actuation coil with a determined surface area *A*. This produces the controllable torque shown in Eq. 2. The normal vector of surface *A* with the vector of *B*_0_ forms the angle *α*:

2 $$\tau _1\left( t \right) = I\left( t \right)A\sin \left( \alpha \right)B_0.$$

The second involves the gradient-switching-produced eddy currents, which lead to a torque $\tau _2\left( t \right)$ as given by Eq. 3:

3 $$\tau _2\left( t \right) = - \frac{1}{8}\dot G_zz\pi \sigma tr_{\mathrm{s}}^4B_0\sin \left( \alpha \right)\cos \left( \alpha \right),$$

where *G*_*z*_ is the gradient slew rate, *z* is the position of the implant (center of gravity), *σ* is the electrical conductivity, *t* is the thickness of the conductive layer, *r*_s_ is the largest dimension of the implant, and *α* is the angle between the normal vector of the implant surface with *B*_0_ and *G*_*z*_ (*B*_0_||*G*_*z*_). As a control group, we prepared an additional set of samples with (i) a sample exhibiting very high conductivity using a 40-µm-thick copper foil (Chomerics CCK-18-101-0200) and (ii) a non-conductive empty PMMA substrate (Fig. 3).

### MR-induced current and voltage measurements

**Fig. 4:**
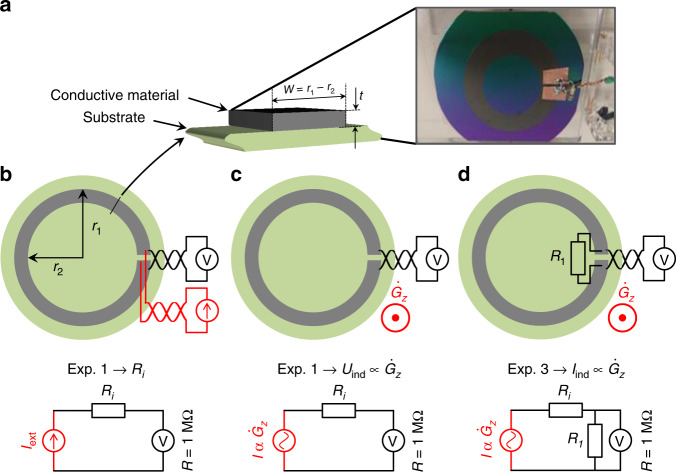
Experimental setup used to investigate the dependence of microelectrode materials on the induced eddy currents. Preparation of conductive rings using Cu (thickness *t* = 40 µm), Pt (*t* = 300 nm), and GC (*t* = 2 µm). The radii are *r*_1_ = 2.5 cm and *r*_2_ = 1.5 cm. **a** Sample on a silicon wafer. **b** Measurement of *R*_i_ by means of four-terminal sensing. **c** Measurement of the induced voltage due to gradient switching in open circuit mode. **d** Measurement of the induced current using a shunt resistor of 1 Ω

To further investigate the performance difference between GC and Pt, we microfabricated open-loop circular dummy microelectrode structures (outer radius = 2.5 cm and inner radius = 1.5 cm) from these two materials, as shown in Fig. 1. The planar geometry for both GC and Pt was the same, with Pt ≈300 nm thick and GC ≈2 µm thick. For the vibration experiments, we also prepared a highly conductive sample from Cu tape as a control experiment. The open-loop microstructures enabled direct measurement of the induced voltages and currents, as shown in Fig. 4. First, we measured the resistance *R*_i_ of the dummy structure using the four-terminal method (Rhode-Schwarz HM 8112-3). In the next step, we measured the induced voltage due to gradient switching in the open-loop configuration, followed by a current measurement using a shunt resistor of 1 Ω. In these measurements, the surface was placed orthogonal to the gradient field. Direct voltage measurements helped to avoid acoustic coupling.

## Results

### MRI measurement

**Fig. 5:**
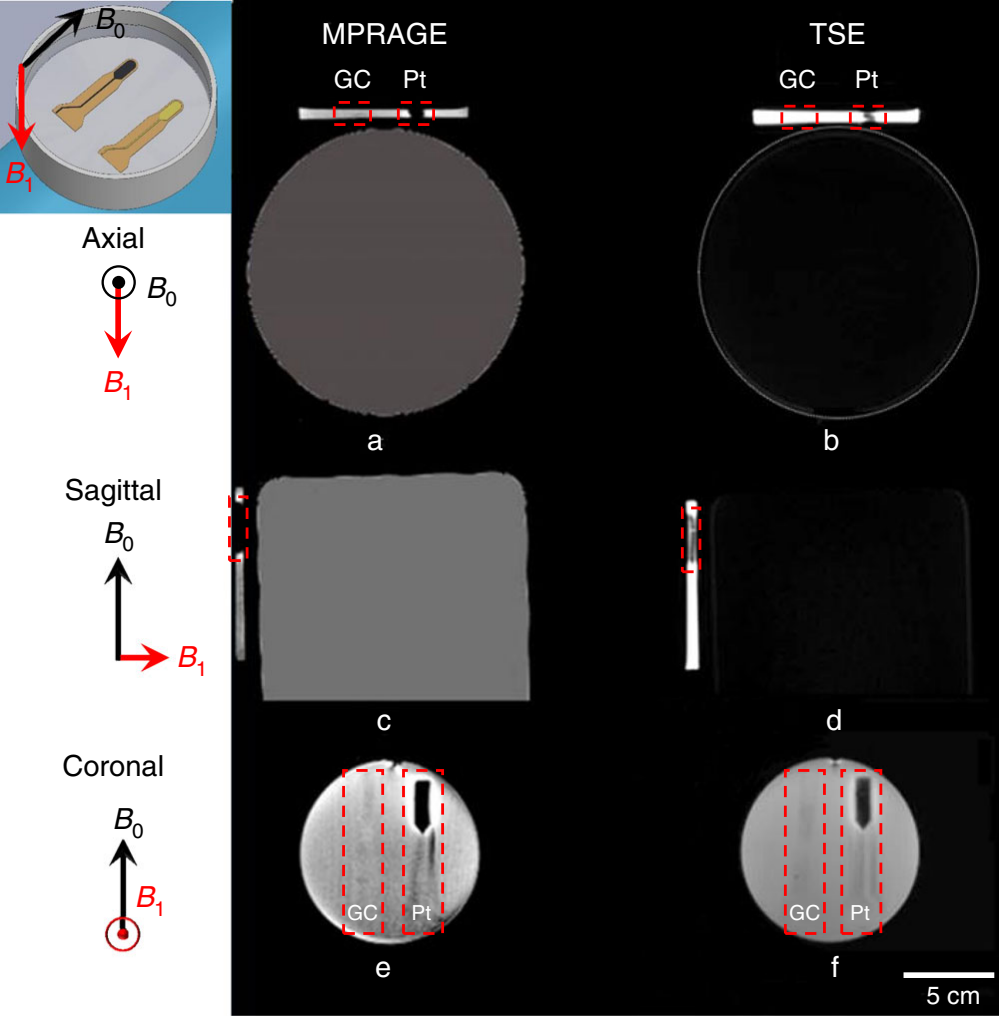
Comparison of magnetic resonance imaging artifact of GC and Pt microelectrode from T1 -weighted and T2-weighted images captured with MPRAGE and TSE sequences, respectively. Pt electrode placed on right side of the agarose phantom showed darkened rectangular shape along the width of Pt electrode in the axial view as compared to the GC electrode (**a**, **b**) for both T1- and T2-weighted images. Similarly, imaging artifact is recorded at Pt electrode along the length in sagittal view of agarose phantom placed on top of MRI phantom (**c**, **d**). In the coronal view, bright spots were observed at Pt and polyimide interface, while no artifacts were observed at GC electrode

The imaging artifacts produced in the 3 T scanner by the GC and Pt microelectrodes were compared in the phantom using clinical MRI sequences. In Fig. 5, the first row shows T1-weighted images taken with the MPRAGE sequence, and the second column shows T2-weighted images taken with the TSE sequence. T1- and T2-weighted images are commonly used in clinical sequences and are represented in axial, sagittal, and coronal views in Fig. 5. The darkened rectangular shape on the right side of the phantom artifact shows loss of signal or hyperintense signals (Fig. 5) corresponding to an imaging artifact produced by the Pt microelectrode. High signals or bright spots are observed at the interface of the Pt microelectrode and the polyimide substrate. Since PBS saline has the same magnetic susceptibility as does brain tissue, it mimics the MR environment of the brain^27^. On the left side of the phantom, an outline of the GC electrode is barely visible (Fig. 5e, f). The Pt electrode sites were ≈300 nm in thickness, but the distortions in MR images observed near the Pt electrode were more spread throughout the thickness of the agarose phantom (4 mm) in the axial and sagittal views (Fig. 5a–d). The MRI in Fig. 5 shows that GC microelectrodes (*χ* = −1.2 p.p.m.^22^) have significantly fewer artifacts than do Pt microelectrodes (*χ* = 279 p.p.m.^27^). Similarly, images captured with the EPI sequence showed loss of signal at the location of the Pt microelectrode, whereas the GC microelectrode position was not differentiable from the phantom background (Supplementary Fig. S6). There is no visible differentiation between the *B*_0_-field maps of the GC and Pt microelectrodes, as shown in Supplementary Fig. S6b.

### High-field MRI

**Fig. 6:**
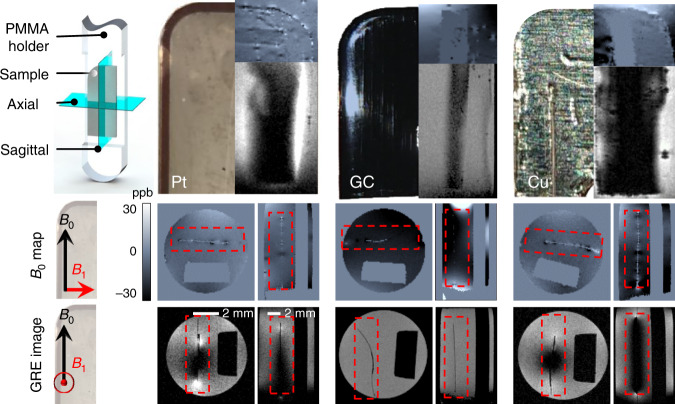
Results of the *B*_0_-field maps and GRE images for the Pt and GC microelectrodes and the copper dummy microelectrode. For the *B*_0_-field map acquisition, the metal samples were oriented such that the *B*_1_ vector is parallel to the electrode surface and orthogonal for the GRE image sequences. For all samples, no significant field distortion is visible. The color map used for the *B*_0_-field maps varies from −30 to 30 p.p.b. (−15 to 15 Hz) (blue encoded images). Slight distortions in the range of a few Hz are visible for the Pt microelectrode. The background field for the GC electrodes shows slight inhomogeneity, but there are no measurable field distortions. Strong *B*_1_-field distortions are observed for the Pt as well as the Cu dummy samples. This is due to the high conductivity of the materials, which yields considerable eddy currents that counteract the *B*_1_ field

The results of the *B*_0_-field measurements and the image experiments conducted in the high-field (11.7 T) magnet are summarized in Fig. 6. The artifacts are mainly due to *B*_1_-field distortion based on induced eddy currents. This conclusion is supported by the fact that there are no severe *B*_0_-field distortions in the *B*_0_-field map, but there are large intensity distortions in the MR images. These intensities are a result of opposing magnetic fields to the *B*_1_-field and induced eddy currents. Compared to the Pt microelectrode, the GC microelectrode sample does not show any image distortions because the smaller conductivity inhibits the formation of considerable adverse eddy currents. This is consistent with what was observed with the 3 T scanner.

### Finite element analysis simulations

**Fig. 7:**
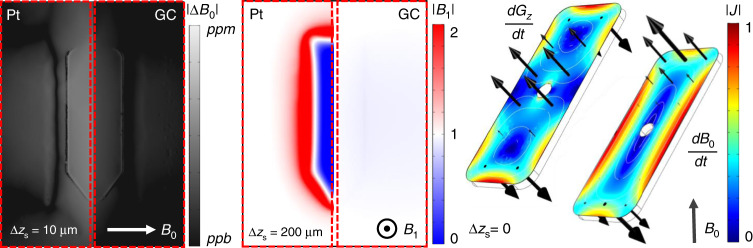
Comparison of the simulated behavior for the 2 cm × 1 cm rectangular GC and Pt ground microelectrodes. On the left, one can see the *B*_0_ relative-inhomogeneity map, at the center, the normalized *B*_1_ inhomogeneity map and, on the right, the induced currents and forces generated by each effect of a unitary gradient’s slew rate, as described above. The results are for a water phantom in a 1.5 T field, shown for varying distances to the electrode surface, Δ*z*_s_, where the effects are at magnitudes relevant to the experiment: 1 p.p.m. for inhomogeneity at Δ*z*_s_ = 10 μm (left image), hundreds of micrometers for a slice thickness (middle image), and current dissipation on the surface of the electrode (right image)

From the results obtained and shown in Fig. 7, it can be observed that a reduced susceptibility mismatch ($\Delta\chi_{\mathrm{H}_{2}\mathrm{O}{\mbox{-}}{\mathrm{GC}}}$ ~=0.04$\Delta\chi_{\mathrm{H}_{2}\mathrm{O}-\mathrm{Pt}}$) and drastically reduced area conductivity (*σ*_tGC_ ~=0.005*σ*_tPt_) lead to a significant reduction in artifacts arising from *B*_1_ and *B*_0_ inhomogeneity. The relative contribution of these will depend on the imaging sequence used. Similarly, the decreased conductivity led to a reduction in the mechanical response coming from gradient switching, in the same ratio as that of the resistances of each microelectrode. Furthermore, due to the reduced conductivity, the microelectrodes have a smaller interaction with the RF fields, meaning the dielectric heating response will be more accurately described by standard RF/SAR simulations, as optimized for safety. Similarly, any Joule heating coming from current dissipation in the microelectrodes will potentially also be reduced by a similarly proportional factor.

When applying a current/force-generating gradient field to the electrodes $B_G = t\left( {\vec G\cdot \overrightarrow {\Delta r} } \right)$, the behavior on a microelectrode can be split into two separate contributions as follows:

4 $$B_G = t\vec G\left( {\overrightarrow {\Delta r} _{{\mathrm{CE}} - G} + \overrightarrow {\Delta r} _{{\mathrm{PE}} - {\mathrm{CE}}}} \right),$$

where $\overrightarrow {\Delta r} _{G - {\mathrm{CE}}}$ is a function of the distance from the center of the electrode to the center of the gradient’s origin and $\overrightarrow {\Delta r} _{{\mathrm{PE}} - {\mathrm{CE}}}$ is the distance from a point in the electrode to its center. The first effect was experimentally measured with the resulting torque but can be fully removed by placing the center of the microelectrode at the gradient’s origin, whereas the second effect cannot be measured and cannot be removed. Given that it is impossible to precisely position an implant in a patient relative to the magnet, introducing a displacement in the cm range, the same order of magnitude of the microelectrode itself, the two effects mentioned will be comparable in magnitude.

### Artifacts measurement: currents

**Fig. 8:**
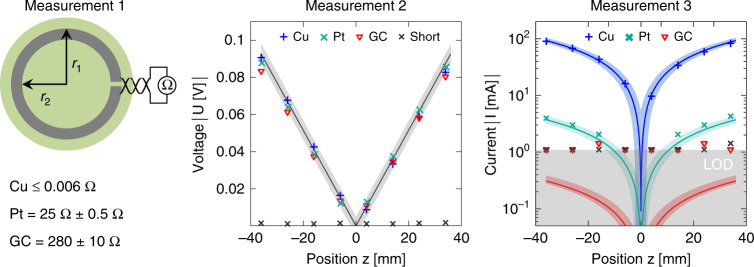
Measurement results from induction experiments. Continuous lines show the interpolated mean values, and the corresponding color-coded areas represent the standard deviation. Actual measurement points are indicated by one set of data points per sample. The first experiment was conducted to measure the resistance of the disc. Middle: The second experiment shows the induced voltage in the rings. The measured values are in good agreement with the theoretical values (solid line). Additionally, short-circuit measurements are presented to highlight the amplitude of erroneous induced voltage in the measurement lines. Right: Induced currents measured with a 1 Ω resistor. The solid lines are the theoretical values obtained using the measured resistances from experiment 1. The limit of detection (LOD) is derived from the deviation of the measurement from the theoretical curve

Investigating the induced voltages in the ring structures allowed for the examination of the gradient field interaction without acoustic noise distortions. Similar to the microelectrode design, the GC and PT samples had the same geometry, except that the thicknesses of the layers were different (Pt ≈300 nm and GC ≈2 µm). The resistances of the test structures were *R*_Pt_ = 25 Ω for the Pt-based microstructure and *R*_GC_ = 280 Ω for the GC microstructure, and *R*_Cu_ ≤0.006 Ω for the Cu tape. As expected, the induced voltage depended only on the surface through which the magnetic flux changed. The theoretical value presented in Fig. 8 is computed using Eq. 5:

5 $$U\left[ V \right] = \pi \left( {r_1^2 - r_2^2} \right)\dot G_zz.$$

The gradient slew rate in this experiment was 2046.6 T/m/s, which leads to the theoretical voltage value displayed in Fig. 8, showing that the measured induced voltage in the rings is in good agreement with the theoretical value (solid line). The measurements with the short end are also presented in the same figure to highlight the amplitude of erroneous induced voltage in the measurement lines. The induced currents measured with a 1 Ω resistor are also given in the same figure, indicating that the current in GC is at least a factor of 10 less than that of the Pt sample. Notably, the current induced in the GC was below the limit of detection (LOD) when using a resistor of 1 Ω.

### Vibration measurements

**Fig. 9:**
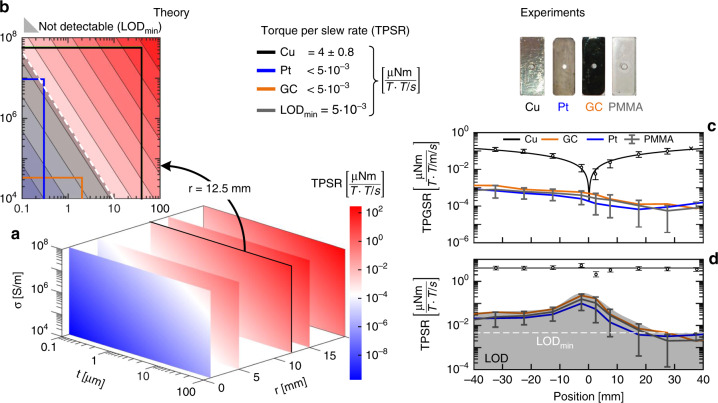
Measurement results of vibration measurements and their comparison to theoretical values. **a** Graph of the torque per unit slew rate (TPSR) as a function of conductivity *σ*, metal layer thickness *t*, and largest dimension size *r*. The value range of the TPSR covers 10 orders of magnitude. **b** A slice of **a** that corresponds to a radius of 12.5 mm. The theoretical values for the tested sites are highlighted, as well as the limit of detection due to superimposed acoustic vibration for implants of this size. The contour lines are per decimal power. The theoretical TPSR for the Pt electrode (blue) and the GC electrode (orange) differ by one order of magnitude (~40×). **c** Graph of the torque per gradient slew rate and field strength that acts on the implants is displayed as a function of the position alongside the bore inside the MR scanner. PMMA samples were used to determine the acoustic-dependent deflection. The vibrations of the Pt and GC samples do not show significant vibrations beyond acoustic-coupled amplitudes. The values are within the standard deviation of the non-conductive samples (error bars for GC and Pt are not plotted here for clarity). The Cu sample, in contrast, shows clear gradient-induced vibration. **d** The torque per slew rate and field strength obtained by normalizing the results from **c** by the position

In Fig. 9, the experimental results are displayed against the theoretical values. The acoustic coupling was measured using empty PMMA plates, which allowed us to determine the LOD. This means that samples with conductive structures are beyond the LOD if their vibration amplitude is not distinguishable from the non-conductive PMMA plates. This is the case for both the Pt and GC microelectrode samples. The sample with copper foil (Cu), in contrast, led to strong vibrational amplitudes of more than two orders of magnitude above the LOD. With this sample, the common mode vibration is clearly detectable where the vibration approaches zero at the gradient center. The acoustic vibration, on the other hand, is strongest inside the MR scanner (140 mm), which leads to the best LOD being achieved at ~30–40 mm. At this point, the gradient slew rate is strongest, whereas the acoustic coupling is minimal. Using theoretical analysis, we can estimate how much the torque of the implants is beyond the LOD. The quality of this analysis is benchmarked by comparing the predicted and experimental values of the Cu sample.

Interestingly, the platinum microelectrode appears to be at the LOD, whereas the GC microelectrode has an approximately 40-fold weaker response (see Fig. 9b). In contrast, the copper foil results in a response three orders of magnitude larger than that of the tested electrodes. We want to emphasize that due to the linearity of electromagnetism, the derived value of torque per unit slew rate may serve as a figure of merit for an implant, which allows a simple derivation of the worst-case torque at any MR scanner. As an example, we may take the Cu foil with a ${\mathrm{TPSR = }}\;4\frac{{{\upmu\mathrm{Nm}}}}{{{\mathrm{T}} \times {\mathrm{T/s}}}}$. Then, in a 3 T human MR scanner with a slew rate of max. 300 T/m/s and largest dimension of 0.3 m (e.g., in the *z*-direction), the maximum expected torque is evaluated as:

6 $$\begin{array}{l}{\mathrm{\tau }}_{\mathrm{max}} = {\mathrm{TPSR}}\times B_{0}\times G_{z}\times z = 4\frac{{{\upmu\mathrm{Nm}}}}{{{\mathrm{T}} \times {\mathrm{T}}/{\mathrm{s}}}}\times 3\,{\mathrm{T}} \times 300\,{\mathrm{T/m/s}} \times 0.3\,{\mathrm{m}} = 1080\,{\upmu\mathrm{Nm}}.\end{array}$$

According to ASTM^28^, critical torque is reached when the MR-induced torque is larger than the gravitational-induced torque. Gravitational-induced torque can be computed using the weight (*m*) of an object multiplied by its longest dimension (*l*). Hence, for the Cu foil, the gravitational torque *τ*_g_ is:

7 $$\begin{array}{l}\tau _{\mathrm{g}} = mgl = 8960\,{{\mathrm{kg}}}/{{{\mathrm{m}}^3}}\times (0.04 \times 25 \times 11)10^{ - 9}\,{\mathrm{m}}^3 \times 9.81\,{\mathrm{m}}/{{\mathrm{s}^2}} \times 25 \times 10^{ - 3}\,{\mathrm{m}} =24.2\,\mu {\mathrm{Nm}} \ll 1080\,{\upmu}{\mathrm{Nm}}.\end{array}$$

Because *τ*_max_ » *τ*_g_, the Cu foil is clearly MR incompatible. On the other hand, the upper limit for the MR-induced torques of the Pt and GC microelectrodes are in the range of 5 × 10^−3^ × 3 T × 300 T/m/s = 4.5 µNm, which is safe.

## Discussion/conclusions

In this work, the MR interactions of GC- and Pt-based microelectrodes were investigated with respect to their adverse effects in producing MR artifacts and gradient-induced vibrations. The specific aim of the MR characterization was to test whether GC microelectrodes used for DBS or the less-invasive intracortical recording (ECoG) produce any artifacts while mapping functional or morphological information of the brain with MR. With regard to gradient-induced vibrations, calculations show that the induced eddy currents are much larger for the Pt microelectrodes than for the GC microelectrodes. However, for both types of microelectrodes, the measurable forces were below the detection limit. In our experimental scanner, the gradient field slew rates are up to a factor of ten larger than in comparable human MRI machines; hence, the expected forces with the presented microelectrodes should remain within a non-hazardous range even in human scanners with up to 7 T. We want to emphasize, however, that the gradient-induced vibration scales to the power of four with the radius of the implants; thus, for larger electrodes, the positive effect of the smaller conductance of GC will be advantageous in obtaining electrode designs that are not prone to vibration induction. We also emphasize the introduction of a new figure of merit that is independent of field strength and gradient slew rate. It provides an important tool for comparing the tendency of different implants to vibrate and enables the straightforward computation for the worst-case torque of an implant for any field strength and slew rate.

MR artifacts, on the other hand, in contrast to vibrations, depend on the conductivity as well as the magnetic susceptibility of the applied material. In summary, our findings show that the larger magnetic susceptibility of platinum does not produce considerable field distortions, especially because of the small volume of the thin-film electrodes. Both GC microelectrodes showed field distortions with <30 p.p.b. at even 11.7 T, and from this viewpoint, both microelectrode types may even allow for use within field-sensitive applications such as localized spectroscopy in human MR machines operating with smaller static magnetic fields (≤3 T). On the other hand, the Pt microelectrode shows considerable *B*_1_-field distortions due to RF-induced eddy currents, which lead to large position-dependent obscuration or hyperintensity. The GC microelectrode is clearly superior with respect to this type of artifact, showing no measurable distortion of the image. The reason is also the much smaller electrical conductivity of GC. It should be noted here that the geometries of these microelectrodes affect their resistance (and hence conductivity), as the resistance *R* is a function of length *L* and cross-sectional area *A* (i.e., *R* = *ρL*/*A* or *R* = *L*/(*σA*)), suggesting that the contribution of geometry alone to the resistance *R* is ~6.6× for Pt compared to GC (since the thickness of Pt = 300 nm and of GC = 2 μm). However, the conductivity (*σ*) of Pt is 9.43 × 10^6^ S/m, which is almost 1400× that of GC (6803 S/m), in effect making the resistance *R* of the Pt microelectrode ~1/200th that of GC. Therefore, despite the smaller thickness, Pt microelectrodes experienced higher induced currents, as demonstrated here. For eddy currents, on the other hand, where the RF resistance is more relevant, length plays a larger role than does thickness due to skin effects; hence, the resistance of Pt and GC microelectrodes will be dominated by their inherent conductivities^29^.

Therefore, we submit that GC microelectrodes demonstrate superior behavior with respect to MR safety compared to Pt-based electrodes. There is strong indication that this statement will be further corroborated by an analysis of RF-induced heating, which was not conduced in this investigation. One large source of RF-induced heating is RF-induced eddy currents; the same currents lead to large MR image artifacts only in the Pt microelectrode and not in the GC microelectrode. We anticipate that the introduced figure of merit will be of great significance as an implant-specific value for labeling torque-related interactions.

Taken together, this study demonstrated that (i) GC microelectrodes experienced no considerable vibration deflection amplitudes and minimally induced currents (below the LOD), while Pt microelectrodes had considerably larger currents, (ii) GC microelectrodes had almost no susceptibility shift artifacts compared to Pt microelectrodes because GC has 1/20th the magnetic susceptibility of Pt and (iii) GC microelectrodes had no eddy-current-induced artifacts, unlike the Pt microelectrodes, mainly because of the lower conductivity of GC (~1/1000th that of Pt) that inhibits the formation of considerable eddy currents. Since GC has recently been demonstrated to have a compelling advantage over other materials for neural stimulation, recordings, and electrochemical sensing of neurotransmitters through voltammetry^4,19^, this MRI compatibility validated in this study offers an additional advantage for long-term in vivo use in clinical settings.

## Supplementary information

Glassy Carbon Microelectrodes Minimize Induced Voltages, Mechanical Vibrations and Artifacts in Magnetic Resonance Imaging
