# Supplementary material for: Glassy carbon microelectrodes minimize induced voltages, mechanical vibrations, and artifacts in magnetic resonance imaging
Source: Microsyst Nanoeng. 2019 Nov 18;5:61. doi: 10.1038/s41378-019-0106-x (PMC6859162; doi:10.1038/s41378-019-0106-x)
Supplement: Supplementary file 1 — Glassy Carbon Microelectrodes Minimize Induced Voltages, Mechanical Vibrations and Artifacts in Magnetic Resonance Imaging [file 41378_2019_106_MOESM1_ESM.docx]

**Glassy Carbon Microelectrodes Minimize Induced Voltages, Mechanical Vibrations and Artifacts in Magnetic Resonance Imaging**

**Surabhi Nimbalkar**^a,d,+^**, Erwin Fuhrer**^b,+^**, Pedro Silva**^b^**, Tri Nguyen**^a,d^**, Martin Sereno**^c^**,**

**Sam Kassegne**^a,d,^**^^[[1]](#footnote-1)^^, Jan Korvink**^b^

^a^ MEMS Research Lab, Department of Mechanical Engineering College of Engineering,

5500 Campanile Drive, San Diego State University, San Diego, CA, USA 92182

^b^ Institute of Microstructure Technology - Karlsruhe Institute of Technology,

Hermann-von-Helmholtz-Platz, 76344 Eggenstein-Leopoldshafen, Germany

^c^ Magnetic Resonance Imaging Lab, San Diego State University, San Diego, CA, USA 92182

^d^ NSF-ERC Center for Neurotechnology (CNT)

*^+^Surabhi Nimbalkar and Erwin Fuhrer contributed equally for this work.*

1. **Microfabrication of Pt microelectrodes on a polymer substrate**


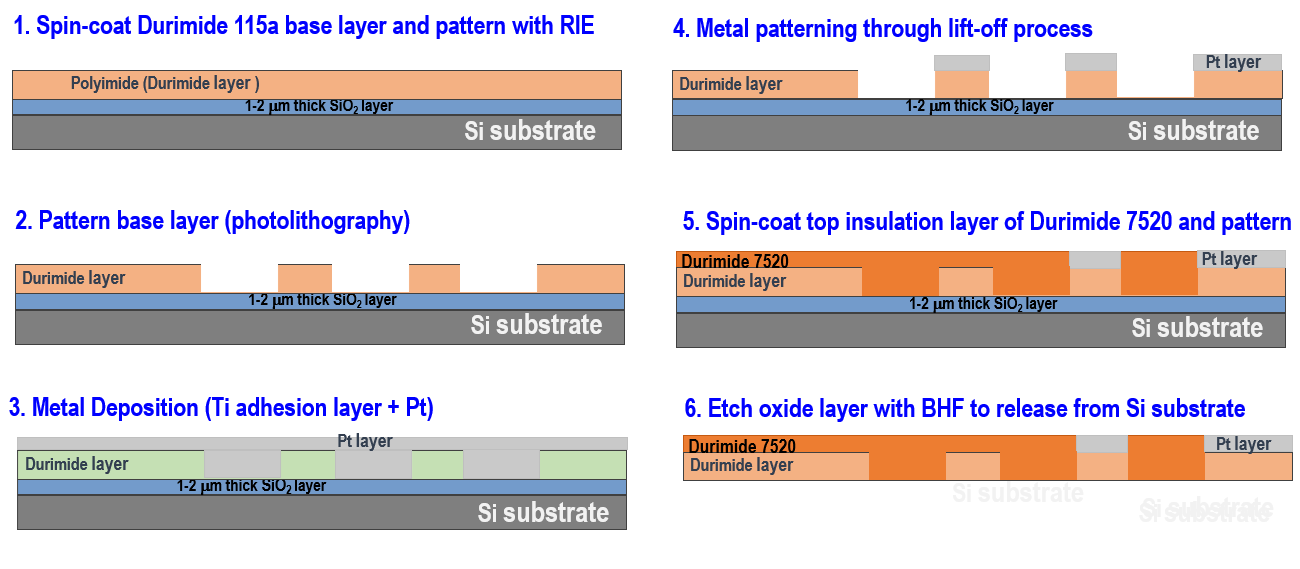


**Figure S.1.** Lithography process for microfabricating Pt microelectrodes on a flexible polymer substrate.

1. **FEA Methods**

The FEM simulations of the effects observed were made in COMSOL Multiphysics (COMSOL AB, Sweden). Static magnetic fields ($B_{0}$ effects) were obtained by solving for the magnetic scalar potential according to Gauss´ law, dynamic electromagnetic behavior (gradient effects) by solving for the magnetic vector potential and current fields according to Ampere’s and Faraday’s laws and the radiofrequency behavior ($B_{1}$ effects) by solving for the electric field in the EM-wave equation.

All simulations were made using the structures shown in a much larger (>10x) volume setting the boundary condition on its outside. These were, respectively, a large box with uniform magnetic flux in one direction on its boundaries, a large box with a time-varying magnetic flux in one direction and an electromagnetic single-loop resonator, excited at the desired frequency, inside a much larger box, at whose boundary the electromagnetic field was null.

The materials simulated were Pt (ε_r_ ~= 0.735, χ = 279 ppm, σ = 9.43x10^6^ S/m), GC (ε_r_ ~= 12.5, χ = -1.2 ppm, σ = 6803 S/m), and PMMA (ε_r_ ~= 2.6, χ = -1.2 ppm, σ ~= 0 S/m) in a water phantom (ε_r_ ~= 80, χ = -9.05 ppm, σ ~= 0 S/m) [21-24].


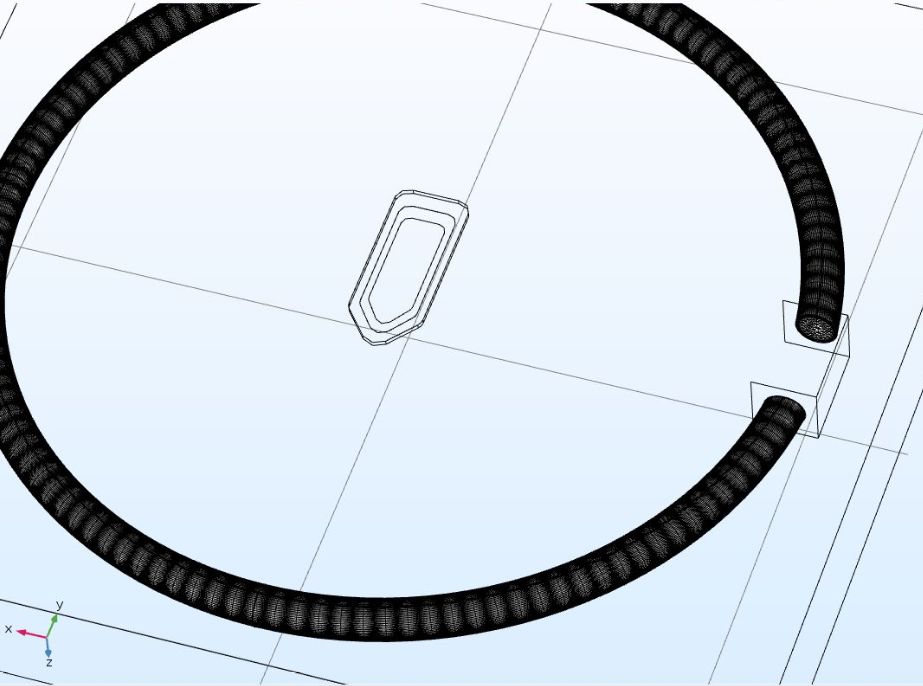


**Figure S.2.** FEA domain used for solving static magnetic effects, magnetic potential vectors, and electric fields.

1. **Three-step measurement protocol to determine the torque produced by the implants**

Connect the actuation coil to the external frequency-controlled voltage supply:

1. Determine the spring constant µ, by sweeping the torque τ_1_ at a constant low frequency (4 Hz).
2. Determine the transfer function and the eigenfrequency of the mechanical oscillation by sweeping the frequency of the torque τ_1_ at constant amplitude.

Disconnect the actuation coil from the external frequency-controlled voltage supply.

1. Application of a defined gradient sequence and measurement of deflection.

We recorded the resulting vibration as well as the excitation pulses to enable a proper analysis in the frequency domain. The used devices are: frequency controlled voltage supply: Tektronix AFG3022B, Acquisition devices: NI-USB9215 & RedLab 1408-FS both connected to the computer via USB readout through MatLab interface.

By measuring the transfer function as explained in step (i) + (ii), one can derive the torque acting on the mechanical device due to the electrode ground planes (see Figure S.2.)

To evaluate and distinguish the effect of acoustic vibration from eddy current induced vibration we run the sequence also with no electrodes attached to the PMMA. Therefore, we tested three different PMMA plates each of them in three different measurement cycles to obtain statistically relevant values about the acoustic coupling. Additionally, we used a sample produced from 40 µm copper foil (Chomerics, CCK-18-101-0200) to evaluate the torque produced by a high conductive sample.

In step (iii) we measure the time domain signal of the gradient switching and the consequential deflection. To measure the gradient switching response of the samples we ran a standard 2D gradient echo sequence (axial slice). To simplify the analysis, we applied a one-dimensional gradient along the z-axis, by setting the field-of-view (FOV) of the two orthogonal gradients to 10 m (at 32 pixels). For the Pt and GC sample we set the slice thickness to 0.4 mm, whereas for the Cu sample to 20 mm. In both cases, the excitation bandwidth was set to 3 kHz. To obtain maximal vibration we excited the corresponding samples on mechanical resonance, thus set $\text{TR}= \frac{1}{f_{res}}$. Using the Fast-Fourier-Transform (FFT) we analyze the signal in the frequency domain which allows to compensate for the mechanical transfer function and to calculate the external torque. From step (i) + (ii) we know the transfer function and the spring constant, and we can derive the torque produced by gradient switching. The results were normalized in reference to the static magnetic field and the gradient slew rate which leads to an implant specific parameter, the position and the field strength we measured. This results in a specific number or figure of merit for an implant namely the specific torque per field slew rate (TPSR $\left[ \frac{Nm}{T·T/s} \right]$). The worst case torque produced by an implant obtained in any MR scanner is obtained by multiplying this number with the slew rate of the target scanner, the maximal possible distance from the gradient center and the corresponding field strength.


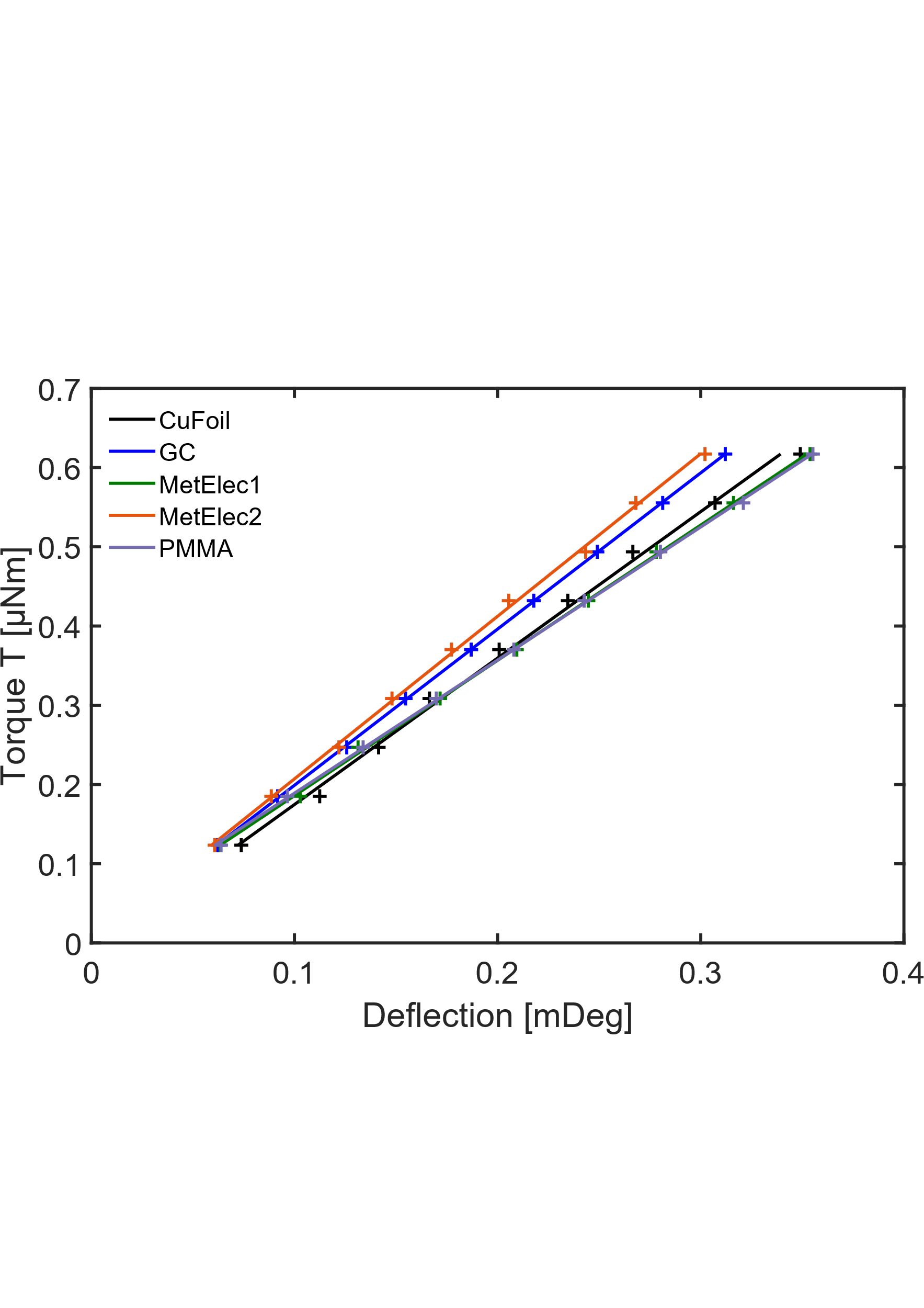


**Figure S.3.** Result of the spring constant measurements of the different samples using the same sample holder. Variations arise due to the maladjustment of the sample holder when exchanging the samples. This leads to a shift of the angle α between the sample holder and the B_0_-field and thus to a different torque (see Equation 2 in the main document). The spring constant of the sample holder is µ = (1.85 ± 0.15) µNm/mDeg.


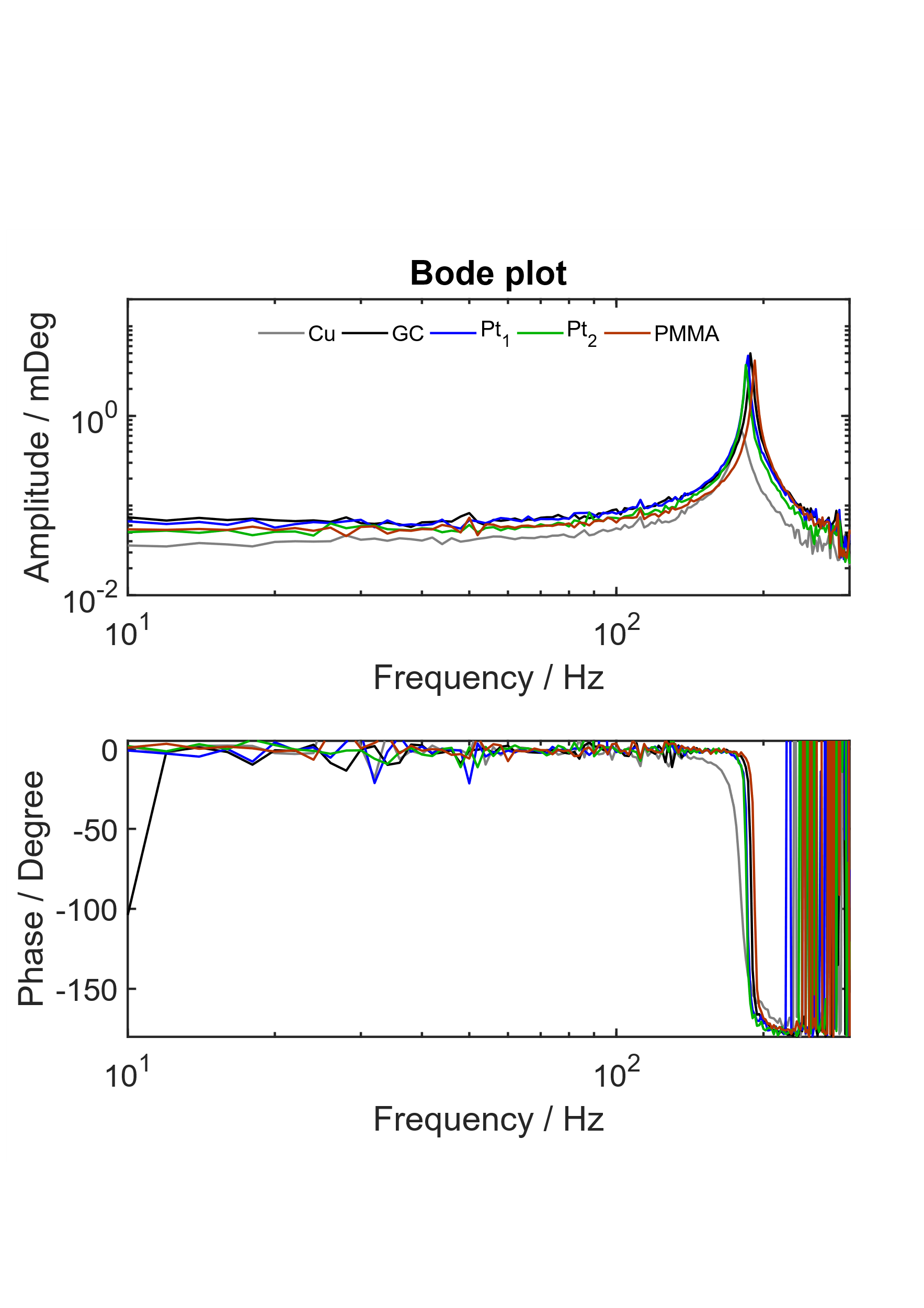


**Figure S.4.** Measured transfer functions of different samples. The harmonic-oscillator-like behavior of the damped spring-mass system is clearly recognizable.


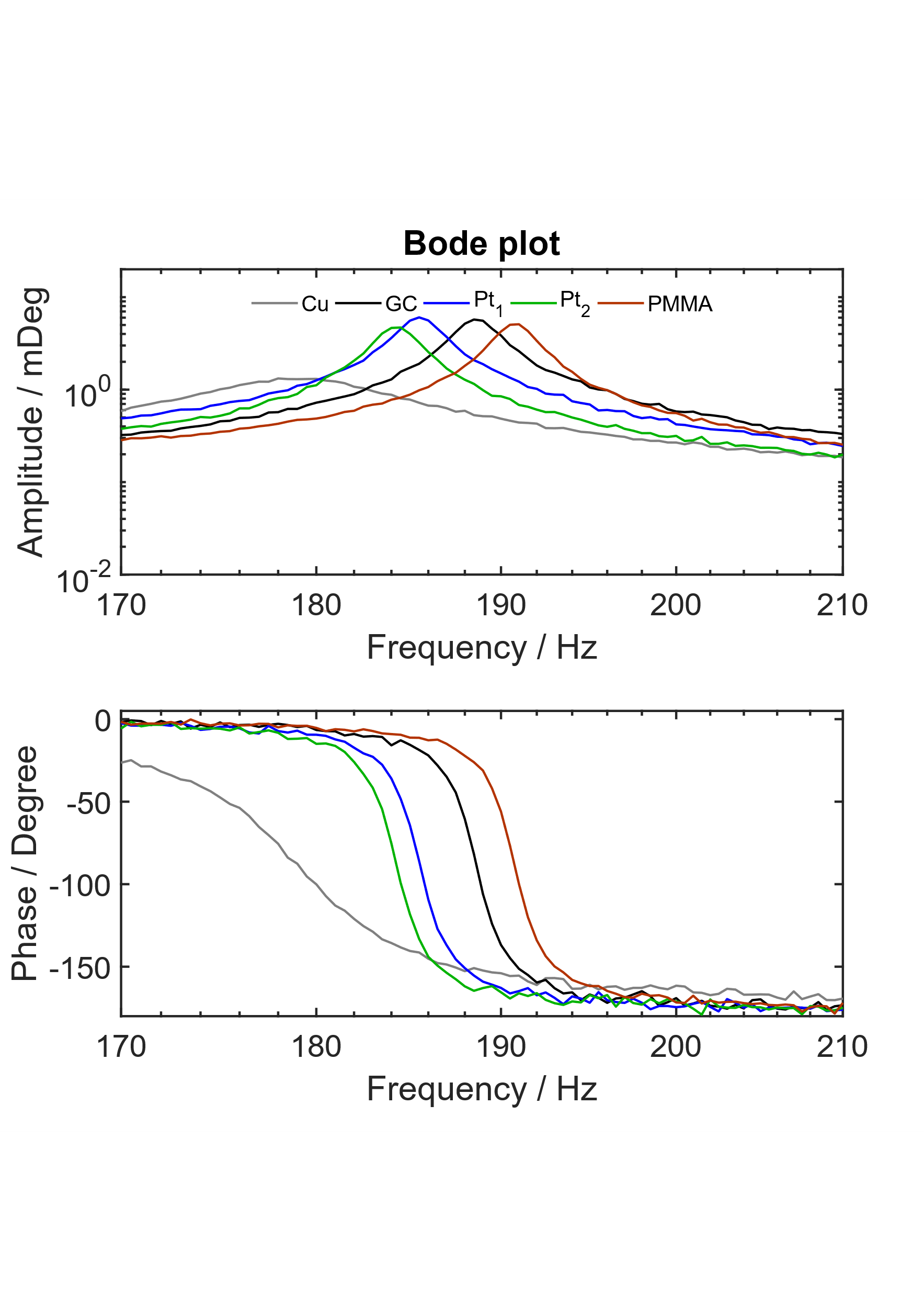


**Figure S.5.** Transfer function with higher frequency resolution around the resonance frequencies. The highest resonance frequency is measured with the PMMA sample holder without an electrode because the moment of inertia is least without an electrode sample attached on the PMMA substrate. The GC microelectrode is lighter than the Pt microelectrodes, hence the resonance frequency of the GC microelectrode is reduced compared to the empty sample holder but larger compared to the Pt microelectrodes. For the copper foil there is, in addition to the reduced resonance frequency, an increased damping observable. The cause is the damping forces due to eddy currents during vibration, induced by the motion of the sample inside the B_0_-field. Hence, the very precise measurement of changes of the transfer function could be used as an examination tool, too.

1. **Imaging Results of B_0_ Field Map and EPI Sequence**


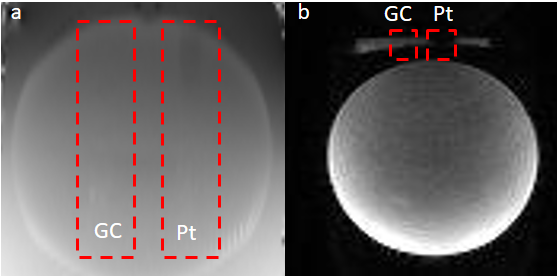


**Figure S.6. (a)** Bo-field map image showing very little or no signal loss at Pt microelectrode site in the coronal view with 3T scanner and **(b)** Echo planar fMRI image showing loss of signal at Pt microelectrode site in axial view with 3T scanner.

1. Address correspondences to Sam Kassegne • Professor of Mechanical Engineering, MEMS Research Lab, Department of Mechanical Engineering, College of Engineering, San Diego State University, 5500 Campanile Drive, CA 92182-1323. E-mail: [kassegne@sdsu.edu](mailto:kassegne@sdsu.edu) • Tel: (760) 402-7162. [↑](#footnote-ref-1)
